# The Impact of Fungal Developmental Structures on Mechanical Properties of Mycelial Materials

**DOI:** 10.1002/elsc.70066

**Published:** 2026-02-24

**Authors:** Kelsey Gray, Harley Edwards, Alexander G. Doan, Walker Huso, JungHun Lee, Wanwei Pan, Nelanne Bolima, Isha Gautam, Tuo Wang, Ranjan Srivastava, Marc Zupan, Mark R. Marten, Steven D. Harris

**Affiliations:** ^1^ Department of Chemical and Biochemical Engineering University of Maryland Baltimore Maryland USA; ^2^ Department of Chemistry Michigan State University East Lansing Michigan USA; ^3^ Department of Chemical and Biomolecular Engineering University of Connecticut Storrs Connecticut USA; ^4^ Department of Mechanical Engineering University of Maryland Baltimore Maryland USA; ^5^ Department of Plant Pathology, Entomology and Microbiology Iowa State University Ames Iowa USA

**Keywords:** asexual development suppression, *Aspergillus nidulans*, cell wall composition, engineered living materials (ELMs), mechanical properties

## Abstract

This study explores how suppressing asexual development in *Aspergillus nidulans* enhances the mechanical properties of mycelial materials. Using four aconidial mutants *(∆brlA*, *∆flbA*, *∆fluG*, and *fadA^G42R^*) lacking asexual development and a control strain (A28) that undergoes typical asexual development, we found that the absence of asexual development significantly improves mechanical strength. All mutants exhibited higher ultimate tensile strength (UTS) than the control, with *∆fluG* and *∆brlA* (fluffy nonsporulating, FNS phenotype) showing the highest UTS. Additionally, *fadA^G42R^* and *∆flbA* (fluffy autolytic dominant, FAD phenotype) demonstrated significantly higher strain at failure (SF), linked to increased autolysis and lower dry cell mass compared to the control and FNS mutants. Solid‐state NMR analysis suggests that autolysis in FAD mutants may disrupt galactofuranose‐related processes, altering cell wall composition and contributing to higher elasticity. These findings suggest suppression of asexual development increases mycelial material strength, while autolysis mechanisms influence elasticity. This research highlights the potential for genetic manipulation in fungi to engineer advanced mycelial‐based materials with tailored mechanical properties.

## 1 Introduction

The urgent need for sustainable materials continues to grow, as environmental concerns escalate and non‐renewable resources become increasingly scarce [1]. Among emerging alternatives to traditional, non‐biodegradable materials, fungal‐based biomaterials stand out for their unique combination of advantages: they are fully bio‐based, cost‐effective to manufacture, and naturally biodegradable [2]. The versatility of fungi enables the production of materials with diverse properties, leading to successful applications across multiple industries, from textiles and packaging to automotive components and building materials [3]. At the heart of these innovations is the mycelium, the vegetative structure of fungi forming intricate networks of microscopic filaments characterized by remarkably efficient growth patterns and robust cell wall architecture [4]. These biological features translate into impressive mechanical capabilities—depending on the fungal species and growth substrate selected, permitting mycelium‐based materials to plausibly match or exceed the mechanical properties of conventional materials [5].

**Practical applications**

This study demonstrates the potential to engineer fungal‐based materials with tailored mechanical properties by suppressing asexual development in *Aspergillus nidulans*. The findings show aconidial mutants produce stronger and, at times, more elastic mycelial materials, making them promising candidates for sustainable alternatives in the packaging, textiles, and construction industries. The increased mechanical strength (e.g., higher ultimate tensile strength) and elasticity (e.g., increased strain at failure) could lead to more durable, biodegradable materials, expanding their applications. Additionally, the link between autolysis and material elasticity suggests further avenues for fine‐tuning fungal biomaterials through genetic modifications. These advancements align with global efforts to reduce reliance on synthetic, non‐renewable materials, offering eco‐friendly solutions with customizable performance. Future applications may include lightweight structural materials, protective coatings, or even medical scaffolds, leveraging fungi's natural growth efficiency and biodegradability.

Recent research has revealed that the mechanical properties of mycelial materials are intricately linked to specific fungal characteristics, including hyphal diameter, cell wall composition, and hyphal packing density [3]. This understanding opens up exciting possibilities for precise material engineering through genetic manipulation of fungi, potentially allowing scientists to fine‐tune these characteristics for specific applications. However, to fully realize this potential, a deeper understanding is needed regarding the complex relationships between fungal genetics, resulting hyphal composition, morphology, and mechanical properties. Such research would enable more precise control over material properties and accelerate the development of next‐generation sustainable materials.

In our previous work, we explored the impact on mycelial‐material mechanical properties of deleting the gene encoding the last protein‐kinase (*mpkA*) in the cell wall integrity (CWI) signaling pathway in *Aspergillus nidulans* [6]. The CWI pathway is responsible for cell wall repair; the deletion of *mpkA* (Δ*mpkA*) leads to significantly altered mycelial phenotypes [7, 8, 9]. Additionally, we have shown previously that Δ*mpkA* mutants have weaker cell walls than an isogenic control when grown in shake flask culture [10]. Because of this, we hypothesized the Δ*mpkA* mutant would produce significantly weaker mycelial material. In contrast, we found the Δ*mpkA* mutant generated significantly (approximately 55%) stronger (ultimate tensile strength, UTS), and more elastic (strain at failure, SF), mycelial material. When characterizing a material's mechanical properties using tensile testing, ultimate tensile strength (UTS) represents the maximum stress endured before fracture, while strain at failure (SF) represents the greatest elongation prior to fracture. To determine the cause of this behavior, we carried out an extensive phenotype analysis of the Δ*mpkA* mutant. Initial analysis revealed a slight difference in the composition between each genotype's cell wall, but the lack of developmental structures observed in the Δ*mpkA* material under scanning electron microscopy was the dominating difference between the morphology of the two materials. This appears to result in more densely packed hyphae and stronger resultant material, as was observed by others, suggesting increased hyphal density produced stronger materials [11].

These morphological observations led us to hypothesize that fungal strains devoid of asexual developmental features would, in‐general, lead to stronger mycelial materials. Asexual development in filamentous fungi is a complex process, regulated by a host of different gene products [12]. When filamentous fungi grow vegetatively, hyphae typically extend at the tip, which is where nutrient assimilation primarily occurs [13]. In response to nutrient deprivation and other signals, these fungi undergo asexual development to produce conidia [14], which serve as protective structures, ready to germinate and grow when additional nutrients become available [15]. Thus, vegetative growth occurs prior to the onset of asexual development [14, 16, 17], creating morphological changes [18, 19], impacting both hyphal biomass and the density of hyphae in a given area [13, 20, 21, 22, 23]. These factors appear to play a role in determining the mechanical properties of mycelial materials [24, 25, 26].

To test the hypothesis that the absence of asexual development leads to stronger mycelial materials, we used the model fungus *A. nidulans*. We chose four different *A. nidulans* mutants (∆*brlA*, ∆*flbA*, ∆*fluG, and fadA^G42R^*) that have previously been shown to not undergo asexual development (i.e., aconidial mutants [27, 28, 29]), and a wild type (A28) control, which undergoes normal asexual development [30, 31]. This group of aconidial, or “fluffy” mutants (based on their appearance when grown on a solid surface), was chosen to broadly cover the aconidial phenotype, rather than to investigate the specific regulatory roles for each of these genes. The goal was to determine the impact of development on mechanical properties rather than to investigate the impact of the gene functions themselves. We note that in the group of aconidial mutants, two phenotypes are represented [32]. The “fluffy non‐sporulating” (FNS) phenotype (i.e., Δ*fluG*, Δ*brlA*) exhibits exclusively vegetative growth, while the “fluffy autolytic dominant” (FAD) mutants (i.e., ∆*flbA*, *fadA^G42R^*) resemble the FNS mutants, with the addition of a pronounced and accelerated autolysis phenotype [32]. Our testing produced data that supports our hypothesis, as we find suppression of asexual development leads to mycelial material with increased ultimate tensile strength (UTS).

## 2 Materials & Methods

### 2.1 Fungal Strains and Growth Media

The *A. nidulans* strains used in this study were all obtained from the Fungal Genetic Stock Center (FGSC, Manhattan, KS, USA) [31] and are listed in Table 1. Initially, strains were grown on modified MAG‐V agar plates (2 g/L BD Bacto Peptone, 1 mL/L vitamin solution, 1 mL/L Hutner's trace elements solution, 20 g/L granulated agar, 1.12 g/L uracil, 1.22 g/L uridine, 35.065 g/L NaCl, 20 g/L glucose, and 20 g/L malt extract [6, 33]). Hutner's trace element solution consisted of 22 g/L ZnSO_4_·7H_2_O, 11 g/L H_3_BO_3_, 5 g/L MnCl_2_·4H_2_O, 5 g/L FeSO_4_·7H_2_O, 1.7 g/L CoCl_2_·6H_2_O, 1.6 g/L CuSO_4_·5H_2_O, 1.5 g/L Na_2_MoO_4_·2H_2_O, and 50 g/L EDTA (Na4). Vitamin solution included 100 mg/L each of biotin, pyridoxine, thiamine, riboflavin, *p*‐aminobenzoic acid, and nicotinic acid [34, 35]. Cultivation of all mycelial materials was in modified YGV medium (1 g/L yeast extract, 2 g/L BD Bacto peptone, 1 g/L Bacto casamino acids, 1 mL/L vitamin solution, 1 mL/L Hutner's trace elements solution, 44.21 g/L KCl, 1.12 g/L uracil, 1.22 g/L uridine, 20 g/L glucose, 20 g/L malt extract, 10 g/L proline, 50 mL/L nitrate salts, and 5 mL/L MgSO4 solution [6, 33]). The nitrate salts were composed of 142.7 g/L KNO_3_, 10.4 g/L KCl, 16.3 g/L KH_2_PO_4_, and 20.9 g/L K_2_HPO_4_, whereas the MgSO_4_ solution required 104 g/L of MgSO_4_.

**TABLE 1:** *A. nidulans* strains used in this study.

Name	FGSC^1^ strain	Phenotype	Genotype	Source
Control	A28	Control	*pabaA6; biA1*	[30, 36]
∆*fluG*	A1828	FNS^2^	*pabaA1; fluG::trpC801; veA1*	[28]
∆*brlA*	A1826	FNS^2^	*argB2; brlA::argB; veA*	[27]
∆*flbA*	A1829	FAD^3^	*biA1; flbA::argB; veA1*	[28]
*fadA^G42R^*	A1827	FAD^3^	*fadAG42R; veA1; biA1*	[29]

^1^ Fungal Genetics Stock Center (Manhattan, KS).

^2^ Fluffy non‐sporulating aconidial strain.

^3^ Fluffy autolytic dominant aconidial strain.

### 2.2 Growing Mycelial Materials

The procedure for generating mycelial material has been described previously [6]. Briefly, fungi were cultivated on modified MAG‐V plates to produce conidia, which were collected, quantified, and used to inoculate YGV medium in a 60 mm Petri dish (Fisherbrand). This was incubated at 28°C for 120 h. After incubation, the grown material disc was harvested and rinsed sequentially with bleach and deionized (DI) water. To prepare material from aconidial mutants, the process began by extracting two “agar cores” from fungi grown on modified MAG‐V plates. These were placed into a cryogenic vial containing 0.5 mL of 3 mm diameter glass beads, and 0.9 mL of DI water that was homogenized for 10 s at 5000 rpm using a Mini Beadbeater (Biospec Products). A 200 µL aliquot of the homogenized solution was then transferred to a 60 mm Petri dish (Fisherbrand) containing 7.5 mL of modified YGV medium and incubated at 28°C for 120 h. The resulting material disc was harvested and washed with bleach and DI water.

### 2.3 Scanning Electron Microscopy (SEM)

Scanning electron microscopy (SEM) was performed following the methodology outlined in [6]. Mycelial samples were examined both prior to and following tensile testing by mounting on an aluminum stub with double‐sided carbon tape, coated with a thin layer of gold/palladium using a Cressington 108 Manual Sputter Coater, and then analyzed using an FEI Nova NanoSEM 450.

### 2.4 Asexual Development (Conidiation)

For quantification of conidia, mycelial material was grown as above, soaked in hexamethyldisilazane (HMDS) for 5 min, then air‐dried for at least 90 min [6]. Dried material was added to a cryogenic vial containing three glass beads (3 mm diameter), and homogenized with a Mini Beadbeater (Biospec Products) at 5000 rpm for 5 s. Then 1 mL of DI water was added, vortexed for 15 s, homogenized again at 5000 rpm for 5 s, and vortexed once more for 15 s. Homogenized samples were diluted with DI water before being vortexed and placed onto a hemocytometer for counting conidia.

### 2.5 Tensile Testing (Mechanical Testing)

Tensile testing was performed on 4 × 40 mm coupons, which were laser‐cut (Universal Laser Systems VLS 3.6/6) from mycelial disks. Five coupons were prepared from each disk, with three disks tested per genotype. Prior to testing, the coupons were immersed in a 40:60 glycerol‐to‐water solution for 1 min [37], coated in petroleum jelly [38], and dried using warm air for about 1 min per side. The dimensions of the coupons were accurately determined using a dissection microscope (Leica S9i, model MEB115), and they were attached to a paper frame for stability using cyanoacrylate adhesive (Loctite) and NaHCO_3_ powder (to speed up drying [39]). The frame was secured to the tensile testing machine (Instron 3369) with double‐sided tape, and just before testing, it was cut on both sides. Reflective tape on the paper frame facilitated strain measurement via a laser extensometer (Electronic Instrument Research Model LE‐01). Stress was recorded using a sensor (Transducer Techniques Sensor) connected to the tensile testing device, which was manually halted after failure [6].

### 2.6 Cell Wall Composition

For cell wall compositional analysis, an entire mycelial material biofilm (prepared as described in Section 2.2) was directly packed into a 3.2 mm rotor, with approximately 33–35 mg of materials were packed in each rotor. All experiments were conducted an 800 MHz (18.8 Tesla) Bruker Avance Neo NMR spectrometer equipped with a 3.2 mm HCN magic‐angle spinning at 290 and 15 KHz MAS. ^13^C chemical shifts were externally referenced to the adamantane CH2 peaks at 38.48 ppm on the tetramethylsilane (TMS) scale. The radiofrequency field strengths for ^1^H decoupling was 83.3–71.4 kHz and 62.5–50 kHz for ^13^C. The proportion of rigid and mobile components was assessed by examining peak volumes in 2D ^13^C‐^13^C spectra, utilizing 53 ms CORD and DP refocused J‐INADEQUATE techniques, respectively [40, 41, 42]. The through‐space CP based CORD spectra were integrated for well‐defined glucan signals that report on the rigid core components, and their relative percentages were calculated. The mobile or flexible components were quantified by integrating the spin‐pair through‐bond correlations obtained from the DP‐INADEQUATE spectra. Peak volumes were quantified using the integration tool in Bruker Topspin 4.1.4 software. To reduce errors caused by overlapping signals, only distinct and well‐separated peaks were included in the analysis following a protocol recently established for fungal cell wall compositional analysis [43, 44]. Due to the biophysical nature of solid‐state NMR, the technique has limited throughput, and consequently only a small number of samples can typically be analyzed. Nevertheless, multiple recent studies on *Aspergillus* and other fungal species have shown that solid‐state NMR spectra are highly reproducible across independently prepared batches [45].

### 2.7 Significance Testing

All data were subjected to T‐tests to identify significant differences between groups using Microsoft Excel. Cell wall composition measurements were normalized to dry cell mass (DCM; i.e., mass of fungi after drying at 90°C for 48 h) for each strain to account for differences in biomass. The normalized data were analyzed using IBM SPSS Statistics (version 30). For each cell wall component (rigid phase: β‐glucan, chitin, α ‐glucan; mobile phase: galactofuranose, galactose, β‐glucan, α‐mannose 1,2, α‐mannose O, α‐mannose 1,6), a one‐way analysis of variance (ANOVA) was performed with strain type as the independent variable and the normalized measurement (Measurement/DCM) as the dependent variable. Homogeneity of variance was assessed using Levene's test. Tukey's Honestly Significant Difference (HSD) post‐hoc test was employed to analyze pairwise differences between fungal strains for each cell wall component. Statistical significance was set at *p* < 0.05.

## 3 Results and Discussion

### 3.1 Fungal Morphology

**FIGURE 1:**
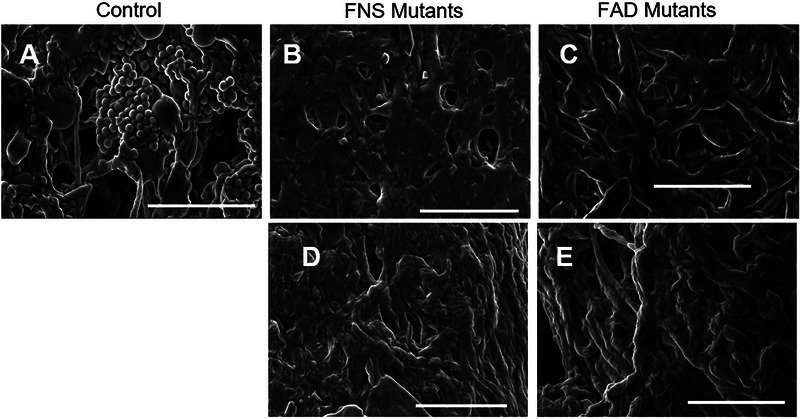
Representative SEM images of mycelial material show 3D structure and morphological features. (**A**) Control (A28) sample surface showing an abundance of developmental structures (e.g., conidia), loose packing of hyphae, and voids in the material. Aconidial mutants: (**B**) ∆*fluG*, (**C**) ∆*brlA*, (**D**) ∆*flbA*, and (**E**) *fadA* ^G42R^ surfaces show more densely packed hyphae, fewer voids, and no developmental structures. All bars = 30 µm.

To confirm the presence or absence of asexual developmental structures (i.e., conidia), mycelial material (*n* = 2 coupons per genotype) was imaged before tensile testing, using scanning electron microscopy (SEM). Figure 1A shows there was an abundance of conidia in material generated from the control strain (A28), but this is not seen in the material generated by any of the four aconidial mutants (Figure 1B–E).

**FIGURE 2:**
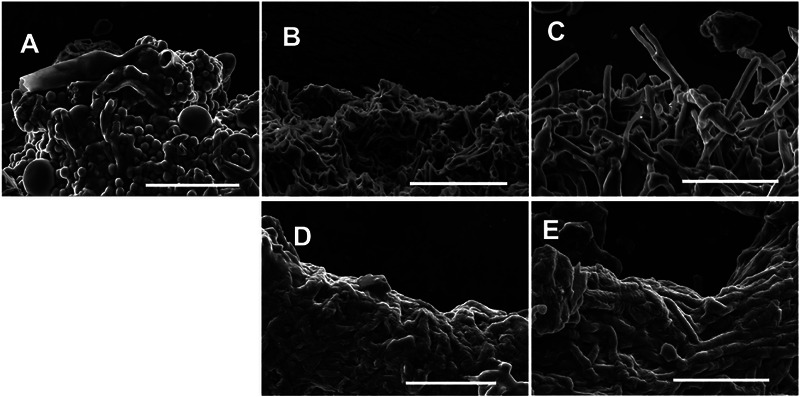
Representative SEM images of mycelial material 3D structure and morphological features at the fracture point after tensile testing. (**A**) Control (A28) sample shows an abundance of developmental structures (e.g., conidia) present. Aconidial mutants: (**B**) ∆*fluG*, (**C**) ∆*brlA*, (**D**) ∆*flbA*, and (**E**) *fadA* ^G42R^ show exclusively hyphal morphology with no developmental structures. All bars = 30 µm.

These images confirm findings from previous studies [46] regarding the degree of asexual development in each of the strains represented. We note that in regard to developmental structures, SEM images of freshly harvested mycelial material (Figure S1) were similar in appearance to those found in the material used for tensile testing. After tensile testing, SEM images of the fracture point for each genotype were collected (Figure 2) and show similar phenomena. The control strain has abundant conidia, while all the other strains do not.

**FIGURE 3:**
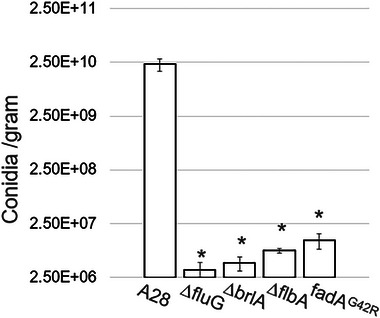
Average number of conidia produced per gram of mycelial material for each genotype tested. Star (*) indicates a significant difference (*P* ≤ 0.05) when compared to control (A28).

Further evidence of the difference in asexual development between the control and mutant strains is provided in Figure 3, where the control (*n* = 2 per genotype) produced on average 2.31E10 conidia/gram, compared to all of the mutants, which produced approximately 10^4^ fewer conidia/g (*p < 0.05*).

### 3.2 Mechanical Properties

**FIGURE 4:**
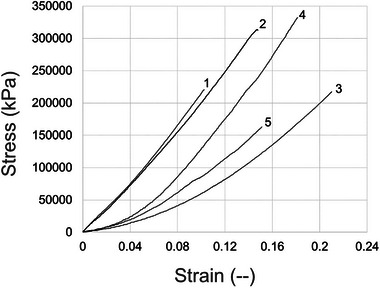
Typical stress‐strain curves for mycelial material generated from the control (A28) strain. Five test strips were cut from the center of a mycelial material disk; numbers on the graph indicate the relative position of strips. This material shows linear elasticity before failure.

To determine material mechanical properties, a mycelial‐material disc was prepared as described above and cut into five strips. Each of these strips was tensile tested, and a stress‐strain curve from a typical test is shown in Figure 4. The numbers in the figure indicate the relative position of the tested coupons cut from the mycelial material disc. Positions 1–5 were immediately adjacent to each other, with position 3 in the middle of the material disk. Similar to the results in Figure 4, all tensile testing showed only random variation with regard to position, implying material was homogeneous with regard to material properties. Representative examples of stress‐strain curves for each of the aconidial mutants are shown in Figure S2.  For each genotype, three mycelial‐material discs were tested (*n* = 15 strips per genotype). In all tests, materials displayed linear elasticity prior to failure, a trait indicative of brittle materials [47].

**FIGURE 5:**
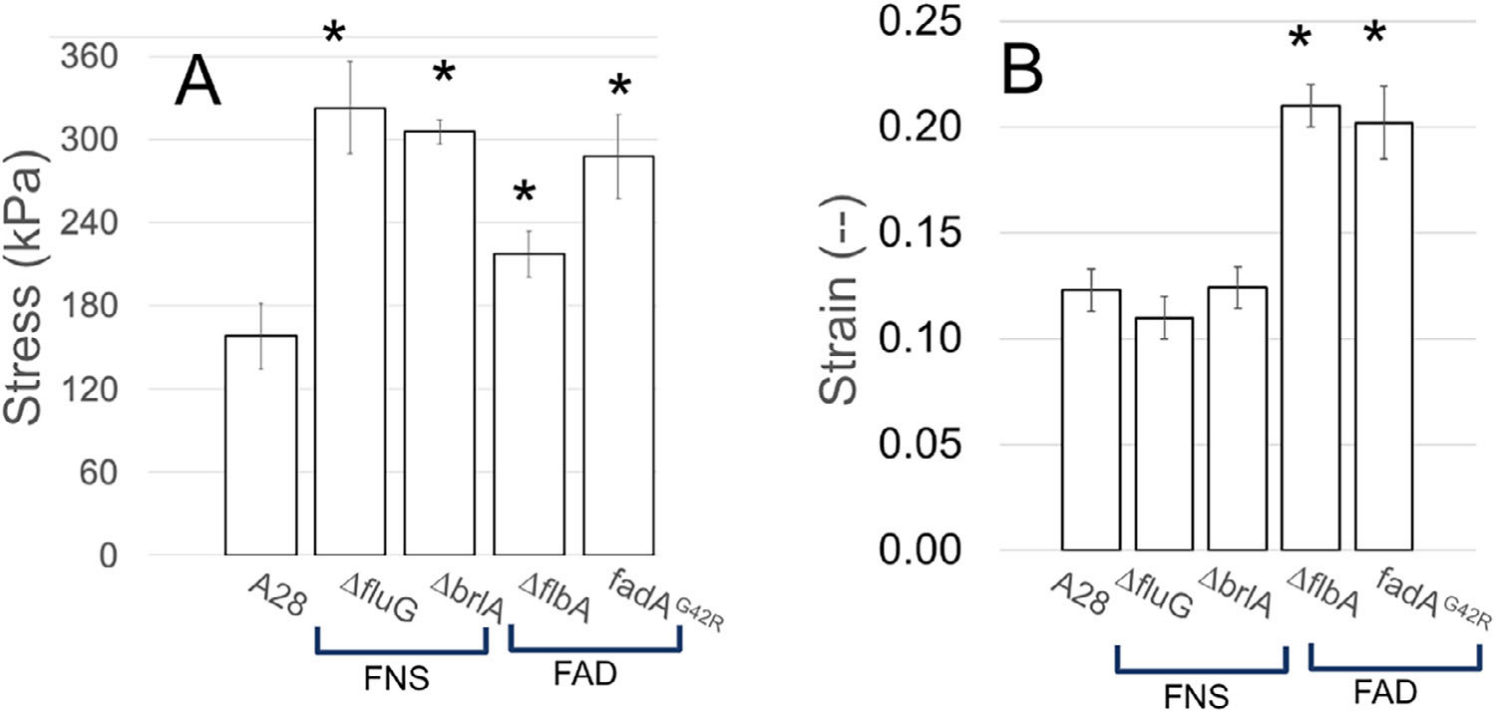
Mechanical properties of mycelial materials. Measurements made during tensile testing (*n* = 15 for each fungal strain) of material generated from control (A28) and aconidial mutants (∆*brlA*, ∆*flbA*, ∆*fluG*, and *fadA* ^G42R^). Averages of (**A**) ultimate tensile strength (stress kPa), and (**B**) strain at failure (–). Star (*) indicates a significant difference (*P* ≤ 0.05) when compared to control (A28).

Data collected from the tensile testing was used to determine: ultimate tensile strength (UTS), and strain at failure (SF). Average values for each of these properties are shown in Figure 5. Figure 5A shows that compared to the control (UTS of 158 ± 24 kPa), all mutants, ∆*fluG*, ∆*brlA*, ∆*flbA*, and *fadA^G42R^*, have significantly higher (*P* ≤ 0.05) UTS of 323 ± 33, 306 ± 8, 218 ± 17, and 288 ± 30 kPa, respectively. Our data show that the aconidial mutants are mechanically stronger than the control, which suggests that asexual development weakens the mechanical strength of mycelial materials. Figure 5B shows that the control, with an SF of 0.12 ± 0.01, was statistically similar (*P* ≥ 0.1) to each FNS mutant, ∆*fluG* with an SF of 0.11 ± 0.01 and ∆*brlA* with an SF of 0.12 ± 0.01. In contrast, the FAD mutants, *fadA^G42R^* and ∆*flbA*, showed significantly higher SF when compared to the control, with an SF of 0.20 ± 0.02 and 0.21 ± 0.01, respectively (*P* ≤ 0.05).

Our work evaluates the mechanical properties of mycelial materials generated from aconidial fungal mutants and finds that the inhibition of asexual development appears, in general, to increase the mechanical strength of mycelial materials. Our data suggest that the phenotypes of the aconidial mutants may have resulted in a hierarchy of strength among mycelial materials, with FNS mutants producing the strongest materials, followed by the FAD mutants, and finally, the control. Mycelial materials generated from the FAD mutants were found to be the most elastic based on their increased strain at failure (Figure 5B). Considering that a major phenotype expressed exclusively among the FAD mutants is their significant degree of autolysis [32], we hypothesized that the accelerated autolysis occurring in these strains may have contributed to altered mechanical properties.

### 3.3 Confirmation of Autolysis and Impact on Cell Wall Composition

**FIGURE 6:**
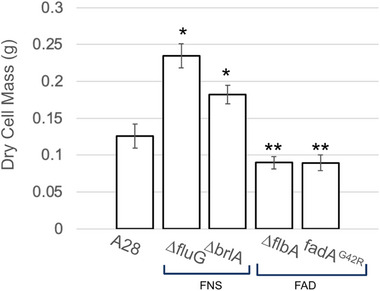
Average dry cell mass of mycelial materials generated from each genotype. Star (*) indicates a significant difference (*P* ≤ 0.05) when compared to control (A28). Double star (**) indicates significant difference (*P* ≤ 0.05) when compared to control and FNS mutants (*∆fluG* and *∆brlA*).

The degree of autolysis exhibited in all strains was assessed by comparing the dry cell mass (DCM) of the mycelial materials generated for each genotype. We hypothesized that strains with the autolytic phenotypes (i.e., FAD mutants) would show a reduced DCM, consistent with previous findings [48]. The results in Figure 6 show this is the case. The FAD mutants have significantly lower DCM when compared to either the control or the FNS mutants, despite the FAD mutants having a hyphal‐dominant morphology, which typically leads to increased amounts of biomass [16]. The significantly reduced biomass in both FAD mutants suggests that accelerated autolysis may be the phenotypic mechanism for their increased strain at failure (SF) observed during mechanical testing.

**FIGURE 7:**
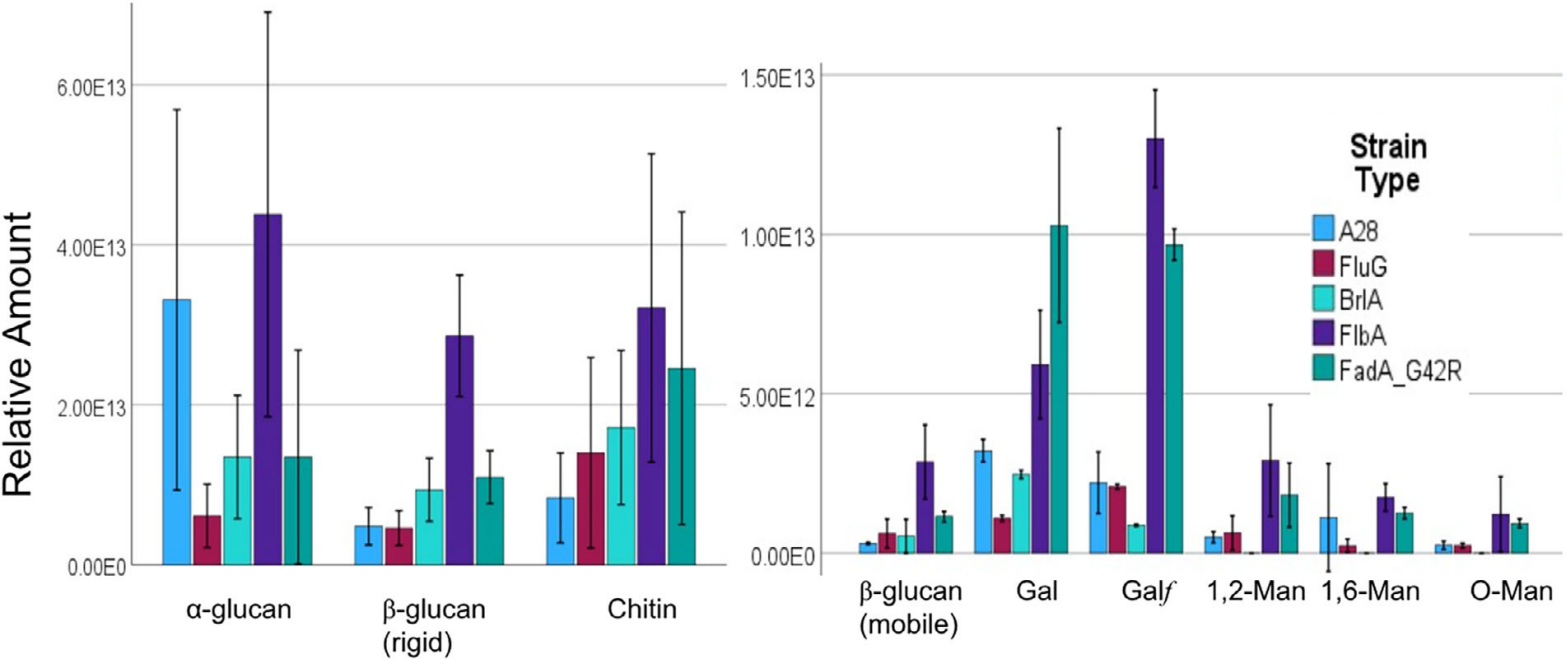
Results from ssNMR cell wall analysis. Left: Rigid phase, Right: Mobile phase. Significance testing is described in the text. Gal in the label represents Gal*p*.

To determine if autolytic degradation in FAD mutants led to altered cell wall composition, we used a high‐resolution, solid‐state NMR approach we have used previously [6], which assessed the composition of both the rigid‐core and mobile‐domain of the cell wall (Figures S3 and S4). Measurements were normalized by DCM (Figure 6), and results are shown in Figure 7. While there is significant variation in the data, statistical analysis of normalized cell wall components provides insight and reveals distinct patterns of alteration across fungal strains. For example, in the rigid phase, the ∆*flbA* mutant exhibited significantly higher β‐glucan content compared to all other strains (*p* < 0.001), with levels approximately 4‐5‐fold higher than the control strain. It is possible that this may play a role in the higher strain at failure values for this strain. Neither chitin nor α‐glucan showed statistically significant differences between strains, though ∆*flbA* displayed numerically higher values for both components.

In the mobile phase, component‐specific effects were observed across mutant strains. Galactofuranose (Gal*f*), a five‐ringed polysaccharide suggested to be required for complete maturation of *Aspergilli* cell walls during hyphal extension [49], presented significantly elevated levels in both FAD mutants compared to the control and FNS mutants, with ∆*flbA* exhibiting the highest levels (*p* < 0.001), followed by *fadA^G42R^* (*p* < 0.001). The content of the mobile phase β‐glucan was significantly higher in ∆*flbA* compared to A28, ∆*brlA*, and ∆*fluG* (*p* < 0.05). Interestingly, for galactose (Gal), *fadA^G42R^* demonstrated the highest levels, significantly exceeding those in the control and FNS mutants (*p* < 0.01). The mannose derivatives (1,2‐Man, O‐Man, 1,6‐Man) displayed limited significant differences between strains.

These results reveal genotype‐specific, rather than phenotype‐specific (i.e., FAD, FNS), effects on cell wall composition. The ∆*flbA* mutation consistently displayed increased glucan content in both rigid and mobile β‐glucan phases, while also significantly elevating galactofuranose levels. *fadA^G42R^*, despite being categorized with ∆*flbA* as an FAD mutant, demonstrated a more selective effect, primarily increasing galactose‐containing components (galactofuranose and galactose). In contrast, FNS mutants (∆*fluG* and ∆*brlA*) showed minimal alterations in cell wall composition compared to the control strain.

These findings suggest that disruption of the ∆*flbA* gene alters cell wall architecture, which may have been the cause of changes in mechanical properties. The differential effects observed between ∆*flbA* and *fadA^G42R^* indicate these genes may influence cell wall composition through distinct mechanisms, despite belonging to the same regulatory pathway [12]. The component‐specific nature of these alterations highlights the complex relationship between genetic variation and resulting phenotypic changes in fungal cell wall structure.

## 4 Concluding Remarks

Consistent with our initial hypothesis, we found that mycelial materials grown from *A. nidulans* aconidial mutants exhibited statistically significant increases in ultimate tensile strength. Additionally, when compared to the control and FNS mutants, both FAD mutants displayed a statistically significant increase in the strain at failure.

## Funding

Student support for this work was provided by an NIGMS Initiative for Maximizing Student Development (IMSD) Grant (5 R25 GM055036), a NIGMS Graduate Research Training Initiative for Student Enhancement (G‐RISE) Grant (T32‐GM144876), G‐RISE at UMBC awarded in 2021, National Science Foundation (NSF 2006189), and the Department of Defense (DoD) Science, Mathematics, and Research for Transformation (SMART) Scholarship—funded by the OUSD/R&E (The Under Secretary of Defense‐Research and Engineering), and the National Defense Education Program (NDEP) / BA‐1, Basic Research. Solid‐state NMR analysis was supported by the National Institutes of Health (NIH) grant R01AI173270 to T.W.

## Conflicts of Interest

The authors declare no conflicts of interest.

## Supporting information




**Supporting File:** elsc70066‐sup‐0001‐Figures.docx.

## Data Availability

All data from this study will be made available upon request from the authors.
