# Supplementary material for: The Impact of Fungal Developmental Structures on Mechanical Properties of Mycelial Materials
Source: Eng Life Sci. 2026 Feb 24;26(2):e70066. doi: 10.1002/elsc.70066 (PMC12930277; doi:10.1002/elsc.70066)
Supplement: Supplementary file 1 — Supporting File: elsc70066‐sup‐0001‐Figures.docx. [file ELSC-26-e70066-s001.docx]

**Supplemental Figures**


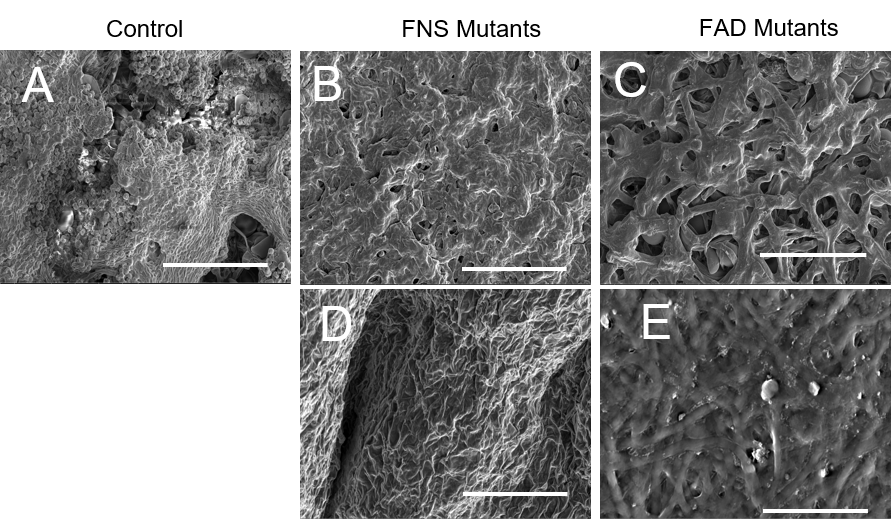


**Figure S1.** Representative SEM images of freshly harvested mycelial material three-dimensional structure and morphological features (some collapsing is present). (**A**) Control (A28) sample surface showing an abundance of asexual developmental structures (i.e., conidia), loose packing of hyphae, and voids in the material. Aconidial mutants: (**B**) ∆*fluG*, (**C**) ∆*brlA*, (**D**) ∆*flbA* and (**E**) *fadA*^G42R^, do not show any aconidial development structures (i.e., conidia). All bars = 50 µm.


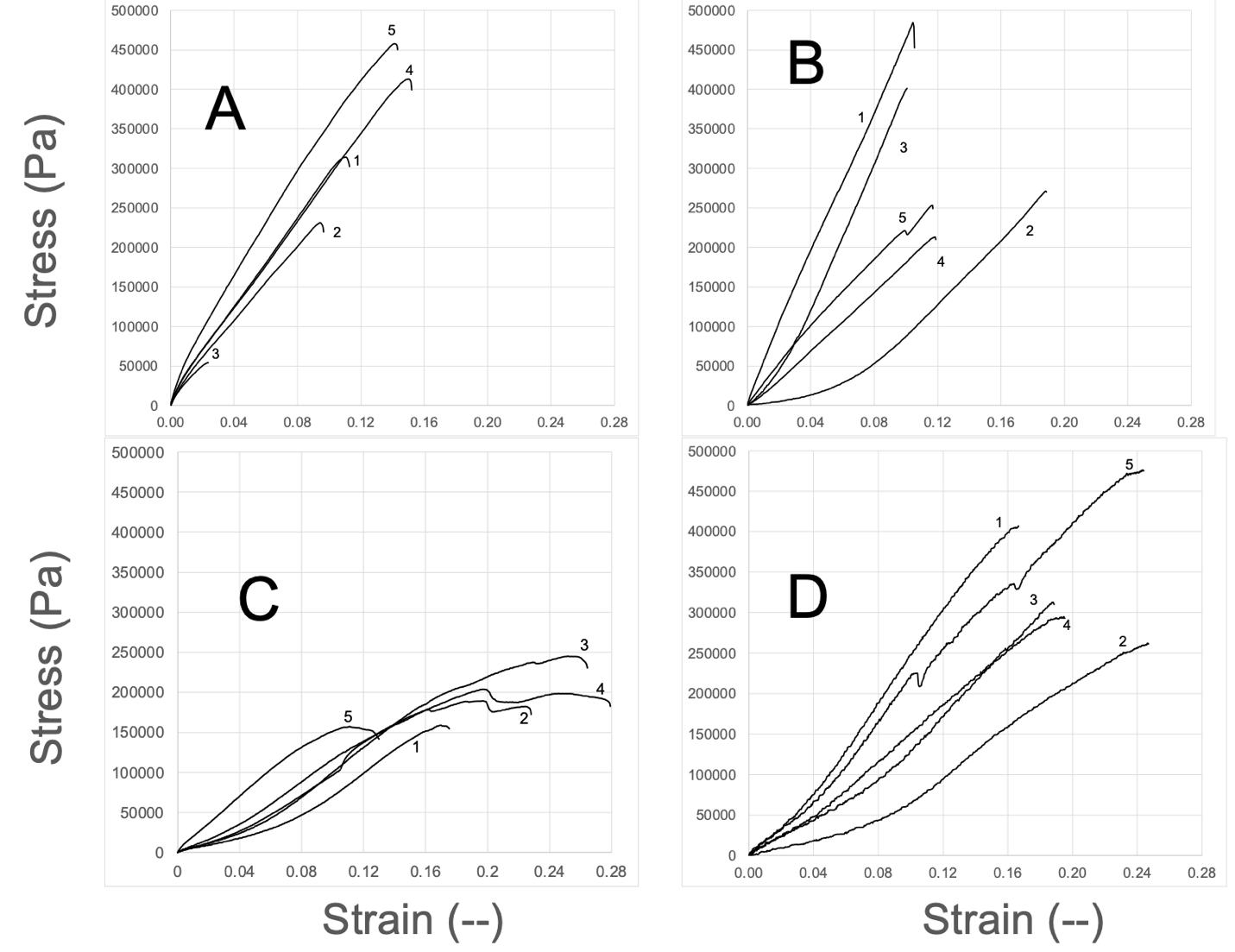


**Figure S2.** Representative stress-strain curve of one mycelial material from each aconidial mutant: (**A**) ∆*fluG*, (**B**) ∆*brlA*, (**C**) ∆*flbA*, and (**D**) *fadA*^G42R^.


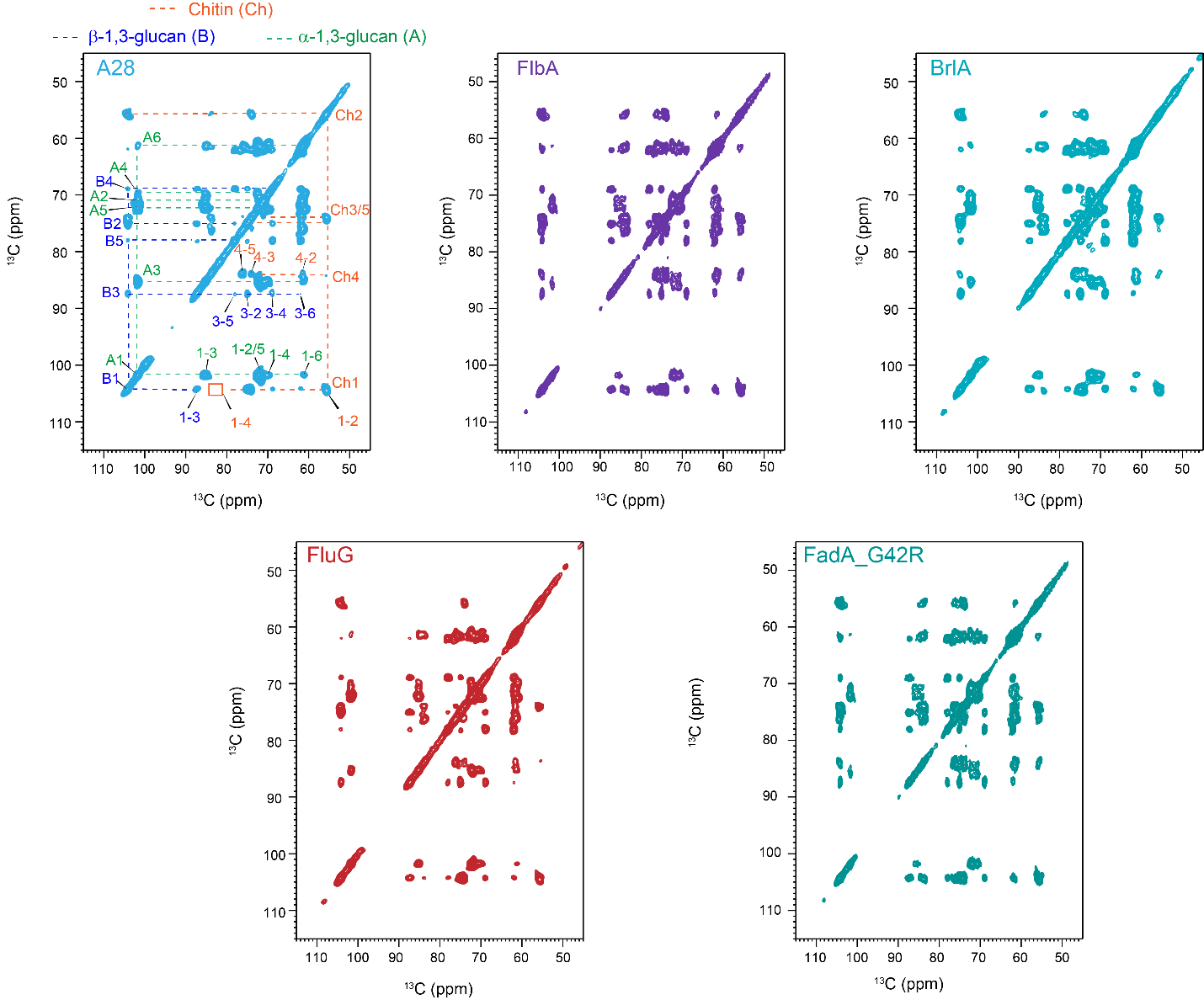


**Figure S3.** Representative change in the rigid glucans in A28, ∆*flbA*, ∆*brlA*, ∆*fluG*, and *fadA*^G42R^ proved by the 2D ^13^C-^13^C correlation spectrum acquired with 53 ms of mixing time. Orange, blue and green dash lines trace the characteristic carbon-carbon correlations for chitin (Ch), β-1,3-glucan (B) and α-1,3-glucans (A) respectively. Each cross peak represents a through- space bond correlation between two carbons within the glucan type, distinguished by color. For example, the 1-3 cross peak in blue represents correlation between C1 and C3 in β-1,3-glucans. Only the resolved assigned are labeled in the spectra.


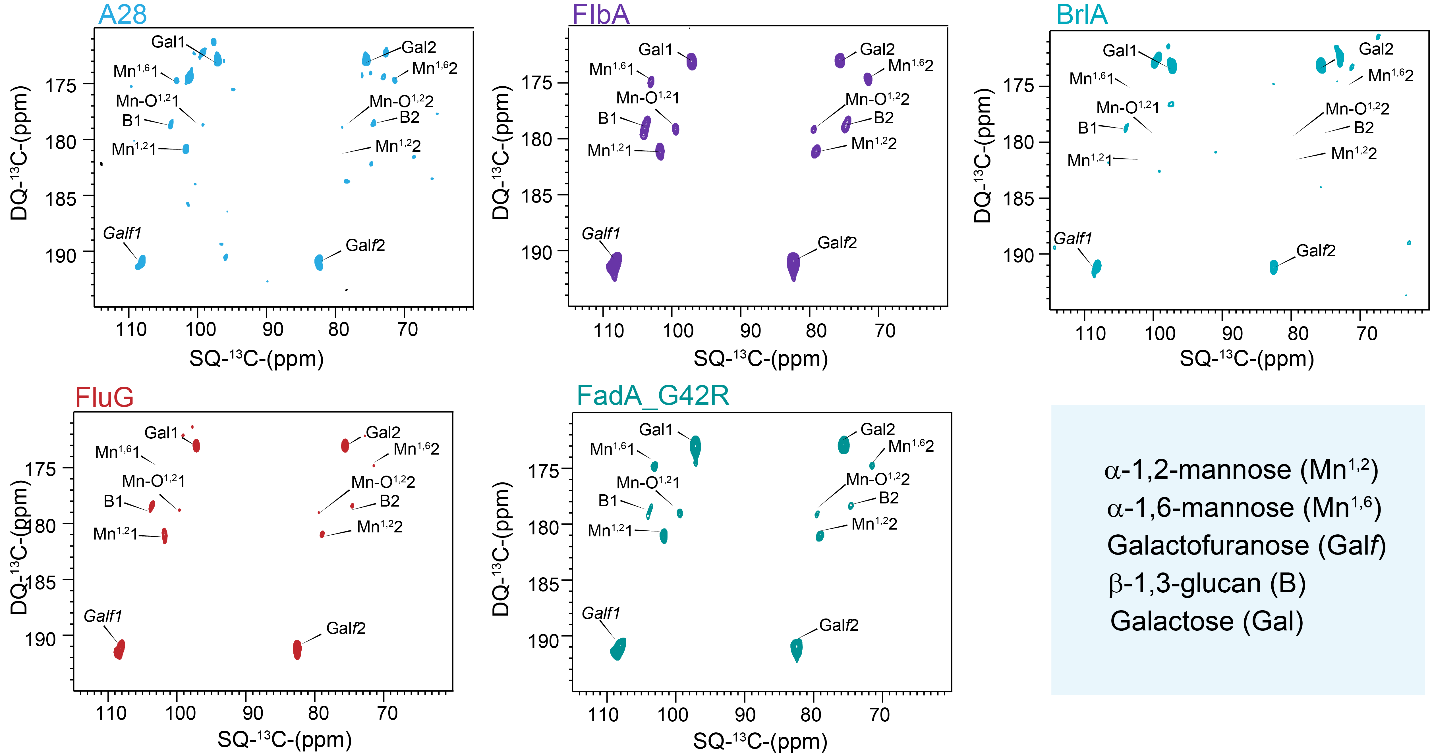


**Figure S4.** Representative change in the mobile glucans in A28, ∆*flbA*, ∆*brlA*, ∆*fluG*, and *fadA*^G42R^ proved by the 2D ^13^C-^13^C through bond DP INADEQUATE experiment. Assignments follow standards NMR abbreviations and carbon numbering for example, Gal*f*1 denotes C1 of galactofuranose, and Gal*f*2 denotes its C2 bond correlation. Only well resolved and unique spin pairs, C1-C2, were used for quantitative analysis. The abbreviations for the mobile glucans included in the analysis are listed in the blue box.
